# Observation of intracranial artery and venous sinus hemodynamics using compressed sensing-accelerated 4D flow MRI: performance at different acceleration factors

**DOI:** 10.3389/fnins.2024.1438003

**Published:** 2024-07-25

**Authors:** Jiajun Cao, Chang Yuan, Yukun Zhang, Yue Quan, Peipei Chang, Jing Yang, Qingwei Song, Yanwei Miao

**Affiliations:** Department of Radiology, The First Affiliated Hospital of Dalian Medical University, Dalian, China

**Keywords:** intracranial artery, venous sinus, compressed sensing, 4D flow MRI, hemodynamic

## Abstract

**Objective:**

To investigate the feasibility and performance of 4D flow MRI accelerated by compressed sensing (CS) for the hemodynamic quantification of intracranial artery and venous sinus.

**Materials and methods:**

Forty healthy volunteers were prospectively recruited, and 20 volunteers underwent 4D flow MRI of cerebral artery, and the remaining volunteers underwent 4D flow MRI of venous sinus. A series of 4D flow MRI was acquired with different acceleration factors (AFs), including sensitivity encoding (SENSE, AF = 4) and CS (AF = CS4, CS6, CS8, and CS10) at a 3.0 T MRI scanner. The hemodynamic parameters, including flow rate, mean velocity, peak velocity, max axial wall shear stress (WSS), average axial WSS, max circumferential WSS, average circumferential WSS, and 3D WSS, were calculated at the internal carotid artery (ICA), transverse sinus (TS), straight sinus (SS), and superior sagittal sinus (SSS).

**Results:**

Compared to the SENSE4 scan, for the left ICA C2, mean velocity measured by CS8 and CS10 groups, and 3D WSS measured by CS6, CS8, and CS10 groups were underestimated; for the right ICA C2, mean velocity measured by CS10 group, and 3D WSS measured by CS8 and CS10 groups were underestimated; for the right ICA C4, mean velocity measured by CS10 group, and 3D WSS measured by CS8 and CS10 groups were underestimated; and for the right ICA C7, mean velocity and 3D WSS measured by CS8 and CS10 groups, and average axial WSS measured by CS8 group were also underestimated (all *p* < 0.05). For the left TS, max axial WSS and 3D WSS measured by CS10 group were significantly underestimated (*p* = 0.032 and 0.003). Similarly, for SS, mean velocity, peak velocity, average axial WSS measured by the CS8 and CS10 groups, max axial WSS measured by CS6, CS8, and CS10 groups, and 3D WSS measured by CS10 group were significantly underestimated compared to the SENSE4 scan (*p* = 0.000–0.021). The hemodynamic parameters measured by CS4 group had only minimal bias and great limits of agreement compared to conventional 4D flow (SENSE4) in the ICA and every venous sinus (the max/min upper limit to low limit of the 95% limits of agreement = 11.4/0.03 to 0.004/−5.7, 14.4/0.05 to −0.03/−9.0, 12.6/0.04 to −0.03/−9.4, 16.8/0.04 to 0.6/−14.1; the max/min bias = 5.0/−1.2, 3.5/−1.4, 4.5/−1.1, 6.6/−4.0 for CS4, CS6, CS8, and CS10, respectively).

**Conclusion:**

CS4 strikes a good balance in 4D flow between flow quantifications and scan time, which could be recommended for routine clinical use.

## 1 Introduction

4D flow MRI, as a kind of novel imaging technique based on three-dimensional time-resolved phase contrast (PC) MRI, has rapidly advanced, with its usefulness as a research tool expanding to a broader clinical role in recent years (Ko et al., 2019; Zhang et al., 2020). By encoding velocity in three directions, 4D flow MRI can retrospectively quantify blood flow at any position within the scanned volume. In addition to the flow rate and velocity obtained by traditional methods like 2D PC MRI, 4D flow MRI can also provide advanced hemodynamic indicators such as wall shear stress (WSS), including axial WSS, circumferential WSS, and 3D WSS, pulse wave velocity, and relative pressure, which are challenging to obtain using other imaging techniques (Björnfot et al., 2021; Kazemi et al., 2022; Ferdian et al., 2023; Park et al., 2023). In this regard, 4D flow MRI has the potential to assist clinicians in the comprehensive assessment of intracranial blood flow (Miller et al., 2019; Marlevi et al., 2021a). While digital subtraction angiography (DSA) remains the gold standard for detecting vascular lesions (Vollherbst et al., 2018), 4D flow MRI demonstrates unique advantages due to its non-invasive, non-radiative nature, and the absence of the need for contrast agents. Studies have shown that 4D flow MRI of intracranial arteries and venous sinuses can guide clinicians in evaluating intracranial aneurysms, plaque, transverse sinus stenosis, multiple sclerosis, venous pulsatile tinnitus, and other neurological conditions (Schuchardt et al., 2019; Ding et al., 2021; Li et al., 2021; Perez-Raya et al., 2021; Zhang et al., 2021; Li et al., 2022). However, these benefits are counterbalanced by long acquisition times, especially when observing intracranial vessels, due to their small lumen diameter and the need for high spatial resolution (Liu et al., 2017).

Due to the sparse and incoherent nature of MR signals, Compressed sensing (CS) could accelerate MRI acquisition through random undersampling of k-space data (Aoike et al., 2022), and then uses nonlinear reconstruction iterative algorithm to reconstruct images with insurance of image quality (Benjamin et al., 2020; Kim et al., 2021). CS has been integrated with parallel imaging (PI) to further expedite flow imaging (Jaeger et al., 2020; Sodhi et al., 2023). Although previous studies have demonstrated the feasibility of CS technology in accelerating the acquisition of 4D flow MRI sequences (Braig et al., 2020; Garreau et al., 2022; Kilinc et al., 2022; Panayiotou et al., 2022), no research has assessed its performance in observing both intracranial artery and venous sinus hemodynamics at various acceleration factors (AFs).

In this work, the performance of 4D flow MRI accelerated by CS technology at four AFs (CS4, CS6, CS8, and CS10) was evaluated and compared with traditional parallel acquisition technology [sensitivity encoding (SENSE, AF = 4)] in a clinical setting. 4D flow MRI of the intracranial artery and venous sinus with these different AFs was used to evaluate the hemodynamics, including flow rate, mean velocity, peak velocity, max axial WSS, average axial WSS, max circumferential WSS, average circumferential WSS, and 3D WSS.

## 2 Materials and methods

### 2.1 Subjects

Forty healthy volunteers were prospectively recruited. The inclusion criteria were: (1) age > 18 years old; (2) No history of brain diseases, including brain tumors, vasculitis, arterial dissection, moyamoya disease, cerebral venous sinus thrombosis, or brain parenchymal trauma, etc., and previously healthy; (3) Able to tolerate MRI scan for approximately 40 min. The exclusion criteria were: (1) Contraindications for MRI scan; (2) Metal implants affecting image analysis; (3) Presence of motion artifacts or poor image quality. Considering the long acquisition time of scanning both arteries and veins, ultimately, 20 volunteers underwent 4D flow MRI of the cerebral artery, and the remaining volunteers underwent 4D flow MRI of the venous sinus. Written informed consent was obtained from all participants. The study was conducted following the Ethics Committee of the First Affiliated Hospital of Dalian Medical University approvals and according to the principles expressed in the Declaration of Helsinki.

### 2.2 Image acquisition

All participants were scanned using a 3.0 T MR scanner (Ingenia CX, Philips Healthcare, Best, Netherlands) equipped with a 32-channel head coil. 4D flow MRI was acquired with different CS acceleration factors (CS4, CS6, CS8, and CS10), while the control scan was acquired with SENSE acceleration factor of 4. The other imaging parameters were: repetition time (TR)/echo time (TE) = 5.1/3.0 ms; flip angle = 8°; field of view (FOV) = 180 × 180 × 46 mm^3^; acquisition voxel size = 1.6 × 1.6 × 1.6 mm^3^; reconstruction voxel size = 0.8 × 0.8 × 0.8 mm^3^; acquisition matrix = 112 × 112; reconstruction matrix = 224 × 224; and velocity encoding (VENC) = 80/60 cm/s (cerebral artery/venous sinus). All 4D flow MRI scans were acquired with retrospective ECG-triggering (with 20 retrospectively reconstructed cardiac phases). The overall scanning time varies with the heart rate of volunteers. Taking heart rate of 90 beats per minute as an example, the scan times for the SENSE4, CS4, CS6, CS8, and CS10 groups were 7 min and 24 s, 5 min and 47 s, 3 min and 52 s, 2 min and 54 s, and 2 min and 22 s, respectively. For heart rate of 70 beats per minute, it took 9 min and 12 s, 7 min and 25 s, 4 min and 58 s, 3 min and 46 s, 3 min and 2 s.

### 2.3 Image processing and analysis

**Figure 1:**
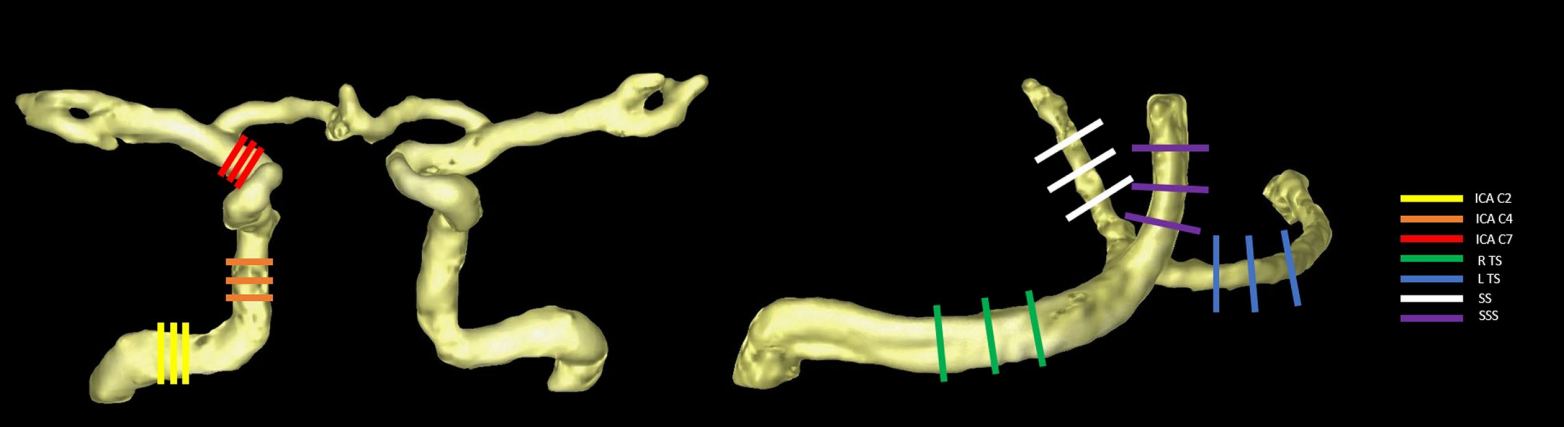
Selected arterial segments (using the right side as an example) **(A)** and venous sinuses **(B)** with measurement planes placed perpendicular to the vessel orientations.

The analysis of hemodynamics in the target blood vessels derived from 4D flow MRI was conducted using the cvi42 software (version 5.14, Circle Cardiovascular Imaging, Canada) by 2 physicians (with 8 and 20 years of experience in MRI diagnosis, respectively). The processing steps included data cropping, preprocessing, vessel segmentation, and hemodynamic measurement. The static tissue and vessel mask were automatically identified and manually adjusted to ensure even distribution across the images. Preprocessing involved offset correction and phase anti-aliasing. To account for the hemodynamic effect of physiological vascular tortuosity, six arterial segments were selected: the left and right internal carotid artery (ICA) C2, C4, and C7 segments (Xie et al., 2024), and four venous sinuses were selected: the left and right transverse sinus (TS), straight sinus (SS), and superior sagittal sinus (SSS). Time-resolved flow quantification was achieved by manually placing three hemodynamic measurement planes orthogonal to the midline of the selected lumens. Subsequently, the contour of the vessel cross-section was automatically drawn and propagated. The contour edge was checked in each time frame, with manual adjustments made if necessary. Finally, the hemodynamics of each vessel were described by the mean of the three planes, including flow rate, mean/peak velocity, average/max axial WSS, average/max circumferential WSS, and 3D WSS (see Figure 1).

### 2.4 Statistical analysis

Statistical analyses were conducted using SPSS version 26.0. Inter-reader measurement consistency was assessed using the intraclass correlation coefficient (ICC). For all hemodynamic parameters, a Shapiro–Wilk test was used to evaluate normality. The results are expressed as mean ± SD if the parameters were normally distributed and as median (interquartile range) if the parameters had a skewed distribution. An ANOVA test was used for normally distributed measurement data and the pairwise differences between sequences were assessed using LSD-t tests. A Friedman Non-normally distributed continuous variables were analyzed using Friedman tests, with pairwise differences evaluated using Wilcoxon tests. Bland–Altman analysis was performed to compare the hemodynamic parameters derived from the 4D flow MRI of the control group (SENSE4) and the CS groups (CS4, CS6, CS8, CS10) respectively. *p*-values for multiple comparisons were adjusted using the Bonferroni correction. The statistical significance level was set at *p* < 0.05, two-tailed.

## 3 Results

A total of 40 participants (25.63 ± 2.71 yrs, female/male, 24/16) were included in the analyses, with 20 participants (25.15 ± 1.31 yrs, female/male, 12/8) undergoing 4D flow MRI of the cerebral artery for hemodynamic analyses of ICA C2, C4, and C7 segments (left and right). The remaining participants (26.10 ±3.58 yrs, female/male, 12/8) underwent 4D flow MRI of the venous sinus for analyses of the transverse sinus (left and right), straight sinus, and superior sagittal sinus. A total of 20 hemodynamic parameters of ICA C2 and C4 segments (left and right), right TS, SSS, 19 hemodynamic parameters of ICA C7 segments (left and right) and SS, and 18 hemodynamic parameters of the left TS were analyzed in the research due to anatomic variations or technical problems. The inter-observer consistency of all hemodynamic parameters of all vessels of interest in both SENSE and CS sequences were all good (all ICC > 0.80, all *p* < 0.05).

### 3.1 Cerebral artery

In the vascular area measurement, for the left ICA and right ICA C7, the area measured by CS10 group were statistically overestimated compared to the SENSE4 scan (*p* < 0.05). For the right ICA C2 and C4, the area measured by CS6, CS8, and CS10 groups were statistically overestimated compared to the SENSE4 scan (*p* < 0.05).

**Figure 2:**
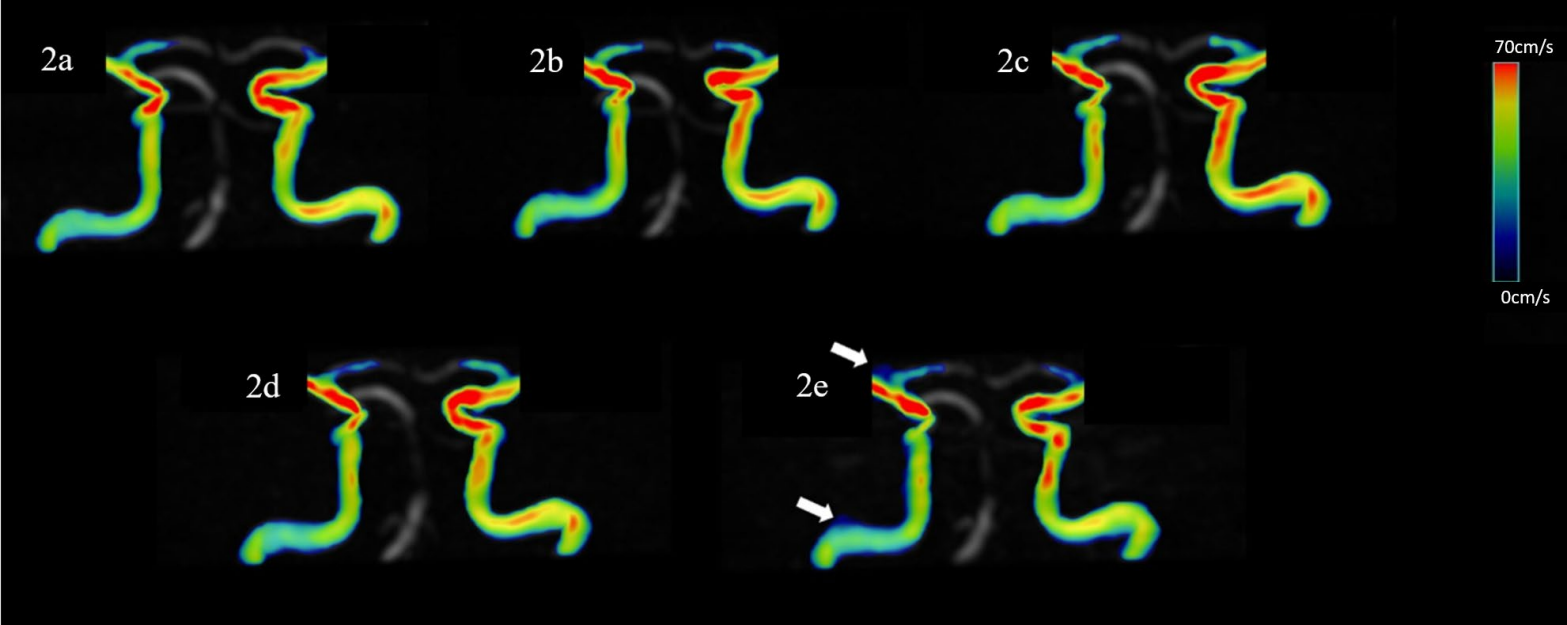
Representative hemodynamics visualization of the ICA with different AFs from the same HC, displaying clear visualization of the straight sinus. Panels **(A–E)** show velocity flow visualization from the SENSE4, CS4, CS6, CS8, and CS10 groups, respectively. In the CS10 group, increased noise around blood vessels is visible (indicated by the white arrow).

No significant difference was observed in all flow quantifications obtained for the left ICA C4 and left ICA C7, as well as the flow rate, peak velocity, average and max axial WSS, average and max circumferential WSS for bilateral ICA C2, the flow rate, peak velocity, max axial WSS, average and max circumferential WSS for the right ICA C4 and C7 among 4D flow MRI scans with different AFs (*p* > 0.05). However, for the left ICA C2, the mean velocity measured by CS8 and CS10 groups, and the 3D WSS measured by CS6, CS8, and CS10 groups; for the right ICA C2, the mean velocity measured by the CS10 group, and the 3D WSS measured by the CS8 and CS10 groups were statistically underestimated compared to the reference (SENSE4) scan (*p* < 0.05). Meanwhile, for the right ICA C4, the mean velocity measured by the CS10 group, and the 3D WSS measured by the CS8 and CS10 groups; and for the right ICA C7, the mean velocity and 3D WSS measured by the CS8 and CS10 groups, and the average axial WSS measured by the CS8 group were also statistically underestimated compared to the reference scan (*p* < 0.05) (see Table 1 and Figure 2).

**Table 1:** Comparison between the flow rate, mean/peak velocity, average/max axial/circumferential WSS, and 3D WSS of cerebral arteries derived from conventional 4D flow and CS-accelerated 4D flows at different acceleration factors.

		SENSE4	CS4	CS6	CS8	CS10
Area (mm^2^)	Left ICA C2	18.81 ± 4.66	22.49 ± 5.09	23.10 ± 7.39	22.95 ± 7.39	25.92 ± 11.33^*^
	Left ICA C4	19.61 (17.09, 23.00)	22.15 (15.04, 27.80)	22.13 (18.37, 24.28)	22.38 (18.22, 27.29)	24.40 (19.61, 26.90)^*^
	Left ICA C7	14.82 ± 4.46	16.51 ± 4.37	16.81 ± 4.65	16.64 ± 5.11	19.09 ± 8.03^*^
	Right ICA C2	17.59 (14.45, 20.05)	20.18 (16.53, 23.03)	21.40 (17.06, 24.71)^*^	21.68 (17.80, 25.00)^*^	25.77 (21.88, 31.74)^*^
	Right ICA C4	16.98 (15.05, 20.80)	19.44 (17.08, 23.55)	21.50 (18.73, 23.71)^*^	20.61 (18.58, 25.30)^*^	23.30 (17.72, 31.42)^*^
	Right ICA C7	12.50 (11.65, 13.62)	13.90 (11.77, 17.82)	14.00 (10.94, 17.41)	14.83 (12.83, 16.46)	15.31 (13.33, 17.01)^*^
Flow rate (mL/s)	Left ICA C2	3.24 ± 1.44	3.61 ± 1.73	3.50 ± 1.56	3.63 ± 1.70	3.44 ± 1.76
	Left ICA C4	5.18 ± 0.69	5.83 ± 0.79	5.50 ± 0.96	5.45 ± 0.93	5.03 ± 1.15
	Left ICA C7	1.86 (1.53, 3.52)	2.49 (1.31, 3.90)	2.46 (1.59, 4.36)	2.40 (1.40, 4.06)	2.37 (1.53, 3.71)
	Right ICA C2	2.97 ± 1.12	3.30 ± 1.33	3.35 ± 1.41	3.11 ± 1.34	3.46 ± 1.48
	Right ICA C4	4.83 ± 0.81	5.38 ± 0.88	5.06 ± 1.00	5.17 ± 0.92	5.13 ± 0.96
	Right ICA C7	2.48 ± 1.21	2.45 ± 1.32	2.49 ± 1.17	2.35 ± 1.05	2.41 ± 1.16
Mean velocity (cm/s)	Left ICA C2	30.40 (27.33, 33.77)	28.90 (24.04, 31.03)	27.85 (24.59, 31.65)	28.42 (25.67, 30.59)^*^	25.65 (22.50, 30.51)^*^
	Left ICA C4	29.46 ± 4.84	27.78 ± 4.28	28.96 ± 4.68	28.27 ± 4.84	27.43 ± 6.54
	Left ICA C7	41.93 ± 7.90	36.89 ± 6.67	38.42 ± 6.37	37.91 ± 6.82	35.62 ± 8.92
	Right ICA C2	30.86 ± 7.03	28.86 ± 6.87	29.29 ± 7.13	26.81 ± 6.46	24.30 ± 6.28^*^
	Right ICA C4	30.74 ± 5.75	28.03 ± 4.93	28.82 ± 4.88	27.77 ± 5.26	26.20 ± 5.17^*^
	Right ICA C7	38.87 (37.02, 43.51)	39.64 (35.33, 41.31)	39.62 (31.56, 44.29)	37.73 (34.82, 43.08)^*^	37.65 (31.63, 42.78)^*^
Peak velocity (cm/s)	Left ICA C2	47.30 ± 7.79	46.85 ± 8.82	46.63 ± 9.65	46.54 ± 9.39	45.87 ± 9.37
	Left ICA C4	44.06 ± 6.43	43.68 ± 6.51	44.58 ± 6.70	45.15 ± 6.37	48.02 ± 6.97
	Left ICA C7	59.35 ± 8.92	57.11 ± 8.59	57.71 ± 8.39	56.92 ± 8.45	56.51 ± 1.00
	Right ICA C2	44.52 ± 9.47	43.50 ± 10.39	44.00 ± 10.05	41.27 ± 9.06	40.70 ± 8.50
	Right ICA C4	45.35 ± 7.70	44.74 ± 8.35	45.05 ± 8.16	45.37 ± 8.00	46.03 ± 8.10
	Right ICA C7	58.41 ± 7.94	56.54 ± 8.00	56.88 ± 7.43	56.45 ± 9.03	58.57 ± 9.36
Average axial WSS (Pa)	Left ICA C2	0.36 ± 0.12	0.38 ± 0.13	0.34 ± 0.10	0.37 ± 0.14	0.33 ± 0.12
	Left ICA C4	0.49 ± 0.13	0.55 ± 0.15	0.50 ± 0.13	0.49 ± 0.13	0.45 ± 0.12
	Left ICA C7	0.49 (0.32, 0.66)	0.57 (0.40, 0.75)	0.45 (0.37, 0.72)	0.44 (0.37, 0.74)	0.40 (0.27, 0.60)
	Right ICA C2	0.29 (0.23, 0.39)	0.32 (0.25, 0.46)	0.32 (0.21, 0.44)	0.31 (0.21, 0.41)	0.28 (0.23, 0.39)
	Right ICA C4	0.55 (0.43, 0.68)	0.47 (0.42, 0.60)	0.53 (0.45, 0.69)	0.57 (0.46, 0.70)	0.60 (0.51, 0.73)
	Right ICA C7	0.56 (0.36, 0.74)	0.51 (0.33, 0.62)	0.44 (0.36, 0.55)	0.44 (0.28, 0.68)^*^	0.46 (0.30, 0.69)
Max axial WSS (Pa)	Left ICA C2	0.71 ± 0.20	0.77 ± 0.24	0.69 ± 0.18	0.73 ± 0.23	0.70 ± 0.22
	Left ICA C4	0.83 ± 0.16	0.96 ± 0.19	0.87 ± 0.17	0.88 ± 0.19	0.87 ± 0.21
	Left ICA C7	0.91 ± 0.37	1.01 ± 0.36	0.97 ± 0.36	0.93 ± 0.26	0.84 ± 0.31
	Right ICA C2	0.55 (0.43, 0.65)	0.64 (0.41, 0.71)	0.59 (0.41, 0.71)	0.57 (0.40, 0.67)	0.53 (0.47, 0.75)
	Right ICA C4	0.91 ± 0.20	1.00 ± 0.22	0.90 ± 0.20	0.95 ± 0.19	0.92 ± 0.15
	Right ICA C7	0.84 (0.69, 1.24)	0.82 (0.58, 1.09)	0.76 (0.60, 0.97)	0.73 (0.55, 1.05)	0.84 (0.50, 0.99)
Average circumferential WSS (Pa)	Left ICA C2	0.35 ± 0.09	0.35 ± 0.09	0.35 ± 0.09	0.35 ± 0.09	0.31 ± 0.08
	Left ICA C4	0.19 (0.18, 0.22)	0.18 (0.16, 0.25)	0.19 (0.17, 0.21)	0.21 (0.18, 0.24)	0.19 (0.17, 0.22)
	Left ICA C7	0.50 ± 0.16	0.53 ± 0.17	0.54 ± 0.19	0.51 ± 0.16	0.49 ± 0.20
	Right ICA C2	0.34 ± 0.09	0.34 ± 0.10	0.34 ± 0.10	0.31 ± 0.08	0.31 ± 0.10
	Right ICA C4	0.18 (0.16, 0.23)	0.18 (0.14, 0.22)	0.18 (0.15, 0.23)	0.19 (0.16, 0.23)	0.17 (0.15, 0.24)
	Right ICA C7	0.51 ± 0.17	0.58 ± 0.22	0.53 ± 0.20	0.53 ± 0.17	0.50 ± 0.17
Max circumferential WSS (Pa)	Left ICA C2	0.80 ± 0.21	0.74 ± 0.15	0.82 ± 0.23	0.82 ± 0.20	0.89 ± 0.29
	Left ICA C4	0.47 (0.44, 0.52)	0.47 (0.41, 0.55)	0.47 (0.41, 0.50)	0.52 (0.44, 0.58)	0.49 (0.41, 0.55)
	Left ICA C7	0.96 ± 0.28	1.02 ± 0.31	1.06 ± 0.33	0.97 ± 0.30	1.00 ± 0.38
	Right ICA C2	0.69 ± 0.17	0.71 ± 0.18	0.71 ± 0.17	0.66 ± 0.15	0.70 ± 0.19
	Right ICA C4	0.44 (0.39, 0.55)	0.43 (0.36, 0.51)	0.43 (0.33, 0.53)	0.44 (0.36, 0.59)	0.40 (0.37, 0.58)
	Right ICA C7	0.96 ± 0.32	1.11 ± 0.41	1.00 ± 0.37	1.01 ± 0.30	0.99 ± 0.34
3D WSS (Pa)	Left ICA C2	0.76 (0.66, 0.91)	0.69 (0.61, 0.84)	0.62 (0.55, 0.76)^*^	0.68 (0.49, 0.81)^*^	0.60 (0.43, 0.74)^*^
	Left ICA C4	0.82 ± 0.87	0.71 ± 0.18	0.76 ± 0.20	0.73 ± 0.21	0.71 ± 0.28
	Left ICA C7	1.03 ± 0.22	0.93 ± 0.25	0.98 ± 0.22	0.95 ± 0.27	0.83 ± 0.30
	Right ICA C2	0.80 ± 0.25	0.71 ± 0.23	0.71 ± 0.26	0.65 ± 0.24^*^	0.52 ± 0.21^*^
	Right ICA C4	0.89 ± 0.23	0.76 ± 0.19	0.80 ± 0.20	0.72 ± 0.23^*^	0.65 ± 0.24^*^
	Right ICA C7	1.10 ± 0.17	1.00 ± 0.18	1.01 ± 0.20	0.94 ± 0.21^*^	0.90 ± 0.21^*^

* There was a statistically significant difference compared with the SENSE4 group.

### 3.2 Venous sinuses

In the vascular area measurement, for the SS, the area measured by CS8 and CS10 groups were statistically overestimated compared to the SENSE4 scan (*p* < 0.05).

**Figure 3:**
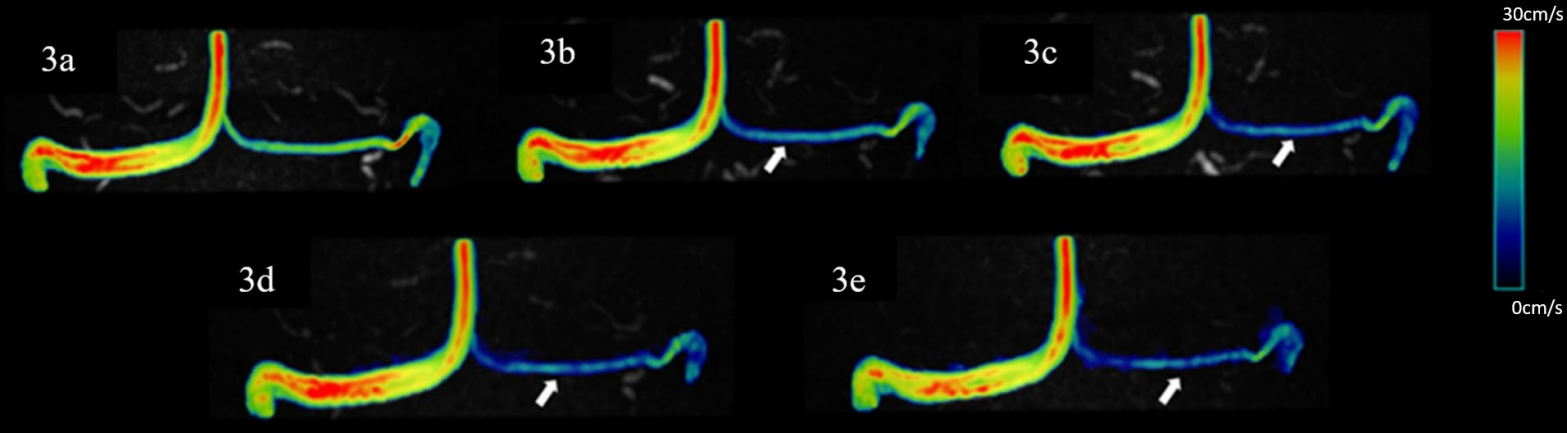
Representative hemodynamics visualization of venous sinuses with different AFs from the same HC, displaying bilateral TS and SSS. Panels **(A–E)** show velocity flow visualization from the SENSE4, CS4, CS6, CS8, and CS10 groups, respectively. There is good similarity between the velocity magnitude distribution in the images, with some underestimation at vessels with smaller diameters (indicated by the white arrow).

**Figure 4:**
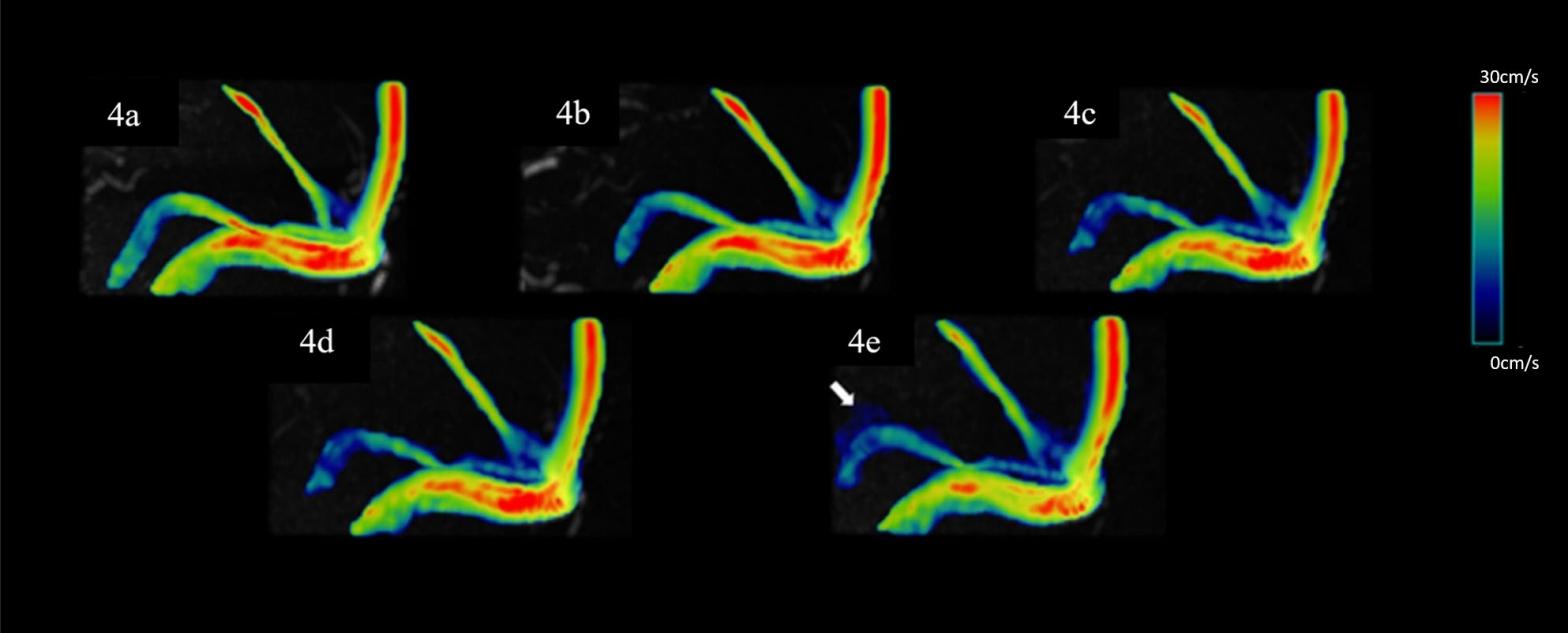
Representative hemodynamics visualization with different AFs from the same HC, displaying the superior SS. Panels **(A–E)** show velocity flow visualization from the SENSE4, CS4, CS6, CS8, and CS10 groups, respectively. In the CS10 group, significantly increased noise around blood vessels is visible (indicated by the white arrow).

No significant difference was observed in all flow quantifications obtained for the right TS and SSS, as well as the flow rate, mean velocity, peak velocity, average axial WSS, and average/max circumferential WSS for the left TS, and the average/max circumferential WSS for the SS among 4D flow MRI scans with different AFs (*p* > 0.05). However, for the left TS, the max axial WSS and 3D WSS measured by the CS10 group were significantly underestimated. Similarly, for the SS, the mean velocity, peak velocity, average axial WSS measured by the CS8 and CS10 groups, the max axial WSS measured by the CS6, CS8, and CS10 groups, and the 3D WSS measured by the CS10 group were significantly underestimated compared to the reference (SENSE4) scan (*p* < 0.05) (see Table 2 and Figures 3, 4).

**Table 2:** Comparison between the flow rate, mean/peak velocity, average/max axial/circumferential WSS, and 3D WSS of venous sinuses derived from conventional 4D flow and CS-accelerated 4D flows at different acceleration factors.

		SENSE4	CS4	CS6	CS8	CS10
Area (mm^2^)	Left TS	24.90 (17.17, 41.20)	26.53 (17.35, 44.58)	23.91 (16.25, 39.86)	22.35 (17.71, 49.61)	28.14 (20.06, 44.83)
	Right TS	33.44 ± 14.49	35.40 ± 15.09	33.47 ± 14.59	35.84 ± 16.24	33.60 ± 14.67
	SS	10.24 (7.90, 14.35)	10.40 (8.87, 13.02)	10.95 (9.03, 15.50)	13.61 (9.76, 17.83) ^*^	13.42 (9.33, 19.79) ^*^
	SSS	31.25 ± 7.71	32.96 ± 8.73	32.42 ± 8.03	30.94 ± 8.17	31.77 ± 10.12
Flow rate (mL/s)	Left TS	3.00 (1.67, 5.85)	2.98 (1.89, 6.02)	2.54 (1.70, 5.95)	2.47 (1.56, 6.18)	2.27 (1.57, 5.56)
	Right TS	3.31 ± 1.52	3.40 ± 1.44	3.21 ± 1.51	3.27 ± 1.56	3.14 ± 1.46
	SS	1.40 (1.35, 1.90)	1.50 (1.28, 1.94)	1.28 (1.18, 1.93)	1.43 (1.13, 1.68)	1.38 (0.97, 1.55)
	SSS	4.72 ± 1.12	5.13 ± 1.09	5.23 ± 1.16	5.05 ± 1.18	4.97 ± 1.12
Mean velocity (cm/s)	Left TS	16.17 ± 3.71	14.92 ± 3.72	15.70 ± 4.37	14.97 ± 3.26	14.23 ± 4.01
	Right TS	17.36 ± 4.77	16.80 ± 4.63	17.33 ± 4.80	16.41 ± 4.45	16.58 ± 4.66
	SS	16.95 ± 3.51	15.90 ± 3.34	14.56 ± 3.58	12.57 ± 3.81^*^	11.93 ± 4.52^*^
	SSS	17.58 ± 4.87	17.68 ± 4.85	18.18 ± 4.70	18.49 ± 5.22	18.17 ± 4.94
Peak velocity (cm/s)	Left TS	26.02 ± 6.00	25.17 ± 6.42	25.74 ± 7.01	24.70 ± 5.58	24.97 ± 7.16
	Right TS	29.62 ± 7.61	28.94 ± 7.36	29.16 ± 7.44	28.65 ± 6.90	28.47 ± 7.75
	SS	26.89 ± 5.47	26.06 ± 5.38	23.99 ± 5.25	22.38 ± 4.75^*^	21.12 ± 5.40^*^
	SSS	30.10 ± 7.27	31.32 ± 7.35	31.48 ± 7.63	31.22 ± 8.31	31.01 ± 7.70
Average axial WSS (Pa)	Left TS	0.27 (0.22, 0.31)	0.25 (0.21, 0.30)	0.24 (0.19, 0.30)	0.23 (0.19, 0.28)	0.21 (0.15, 0.27)
	Right TS	0.26 ± 0.09	0.25 ± 0.08	0.25 ± 0.09	0.23 ± 0.07	0.24 ± 0.08
	SS	0.53 (0.46, 0.55)	0.51 (0.44, 0.56)	0.49 (0.36, 0.53)	0.38 (0.33, 0.46)^*^	0.37 (0.25, 0.41)^*^
	SSS	0.44 ± 0.15	0.44 ± 0.15	0.43 ± 0.14	0.42 ± 0.16	0.41 ± 0.15
Max axial WSS (Pa)	Left TS	0.48 (0.38, 0.53)	0.45 (0.34, 0.49)	0.40 (0.33, 0.52)	0.37 (0.34, 0.49)	0.36 (0.29, 0.47)^*^
	Right TS	0.46 ± 0.14	0.44 ± 0.14	0.43 ± 0.14	0.41 ± 0.12	0.42 ± 0.13
	SS	0.67 (0.57, 0.75)	0.65 (0.60, 0.77)	0.61 (0.50, 0.69)^*^	0.58 (0.48, 0.63)^*^	0.49 (0.36, 0.58)^*^
	SSS	0.73 (0.51, 0.97)	0.75 (0.51, 0.98)	0.75 (0.55, 1.00)	0.64 (0.51, 0.91)	0.58 (0.47, 0.95)
Average circumferential WSS (Pa)	Left TS	0.16 (0.13, 0.19)	0.15 (0.11, 0.19)	0.15 (0.11, 0.20)	0.13 (0.12, 0.19)	0.13 (0.10, 0.19)
	Right TS	0.21 ± 0.08	0.20 ± 0.07	0.20 ± 0.06	0.18 ± 0.06	0.18 ± 0.06
	SS	0.08 (0.06, 0.09)	0.06 (0.05, 0.09)	0.07 (0.05, 0.08)	0.07 (0.06, 0.08)	0.06 (0.05, 0.08)
	SSS	0.08 (0.07, 0.11)	0.08 (0.05, 0.10)	0.08 (0.06, 0.09)	0.07 (0.06, 0.09)	0.08 (0.06, 0.09)
Max circumferential WSS (Pa)	Left TS	0.34 (0.26, 0.41)	0.32 (0.23, 0.39)	0.31 (0.24, 0.44)	0.31 (0.24, 0.43)	0.28 (0.21, 0.44)
	Right TS	0.43 ± 0.14	0.42 ± 0.14	0.43 ± 0.14	0.39 ± 0.12	0.38 ± 0.13
	SS	0.14 (0.11, 0.16)	0.12 (0.10, 0.16)	0.13 (0.11, 0.17)	0.13 (0.12, 0.16)	0.12 (0.10, 0.15)
	SSS	0.19 (0.15, 0.22)	0.19 (0.15, 0.22)	0.17 (0.15, 0.21)	0.17 (0.15, 0.20)	0.18 (0.15, 0.23)
3D WSS (Pa)	Left TS	0.34 (0.26, 0.43)	0.30 (0.25, 0.39)	0.30 (0.25, 0.39)	0.31 (0.23, 0.35)	0.27 (0.23, 0.35)^*^
	Right TS	0.38 ± 0.14	0.36 ± 0.13	0.38 ± 0.14	0.34 ± 0.13	0.34 ± 0.13
	SS	0.43 ± 0.14	0.43 ± 0.13	0.40 ± 0.14	0.36 ± 0.12	0.23 ± 0.13^*^
	SSS	0.39 ± 0.13	0.38 ± 0.14	0.40 ± 0.15	0.40 ± 0.14	0.35 ± 0.13

* There was a statistically significant difference compared with the SENSE4 group.

**Figure 5:**
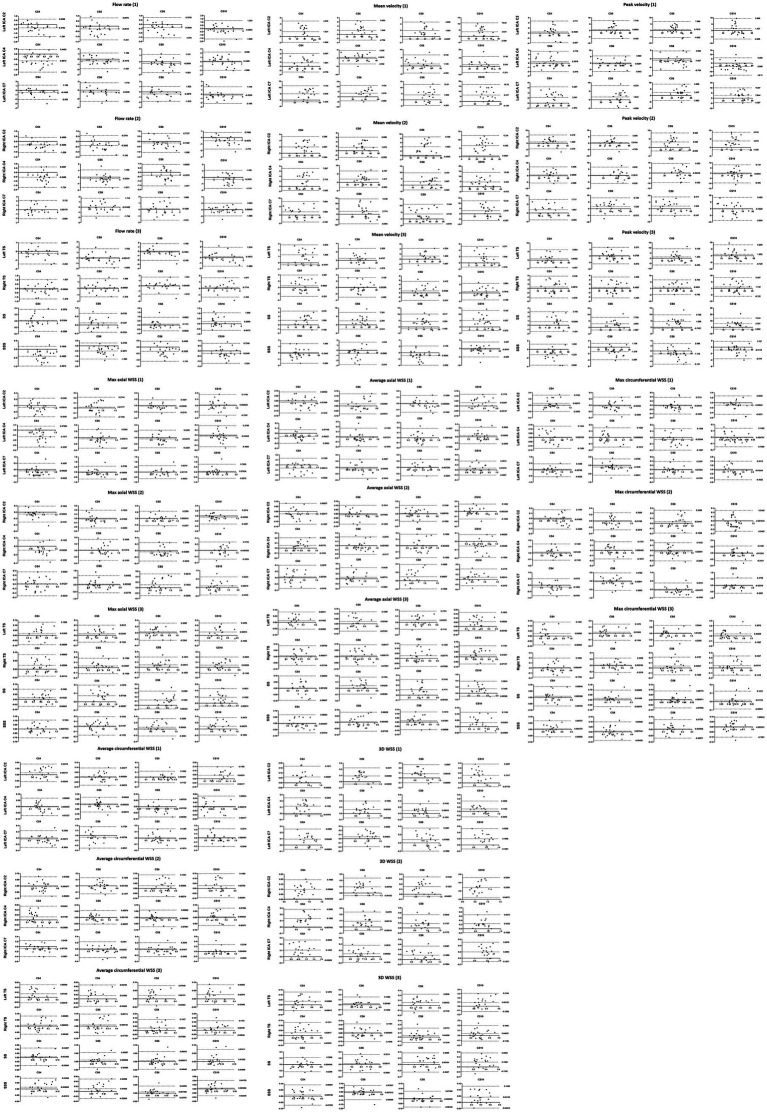
Bland–Altman plots comparing the measurements from the conventional and CS 4D flow scans for the flow rate, mean velocity, peak velocity, max axial WSS, average axial WSS, max circumferential WSS, average circumferential WSS and 3D WSS. The upper line and the lower line: 95% limit of agreement; The middle line: bias.

Bland–Altman analysis revealed that the hemodynamic parameters measured by the CS4 group had only minimal bias and great limits of agreement compared to conventional 4D flow (SENSE4) in the ICA and every venous sinus (the max/min upper limit to low limit of the 95% limits of agreement = 11.4/0.03 to 0.004/−5.7, 14.4/0.05 to −0.03/−9.0, 12.6/0.04 to −0.03/−9.4, 16.8/0.04 to 0.6/−14.1; the max/min bias = 5.0/−1.2, 3.5/−1.4, 4.5/−1.1, 6.6/−4.0 for CS4, CS6, CS8, and CS10, respectively). With the increase of AFs, the consistency of the other groups decreased to varying degrees (see Figure 5 and Supplementary file).

## 4 Discussion

4D flow MRI has emerged as a promising technique for visualizing and quantifying blood flow in the heart and major blood vessels. It enables simultaneous velocity encoding in all three directions and offers 3D volume information resolved over time (Demirkiran et al., 2022). The feasibility of 4D flow MRI in cardiac and macrovascular imaging has been well-established (Moersdorf et al., 2019; Marlevi et al., 2021b). However, neurovascular imaging presents unique challenges due to the complex anatomy and blood flow patterns involved. Previous studies have highlighted the importance of keeping the scanning time of a single sequence within 10 min for clinical acceptance, aligning with clinical workflow and patient comfort (Boussel et al., 2009). However, the scanning time of non-accelerated 4D flow MRI for intracranial vessels often exceeds this limit, making it impractical for clinical use. CS technology offers a solution by significantly reducing scan time while preserving image quality (Meister et al., 2023; Sui et al., 2023; Zhang et al., 2024). A study focusing on hemodynamic analysis of intracranial aneurysms (IAS) demonstrated that 4D flow MRI accelerated by CS provided comparable qualitative and quantitative evaluation to the reference (accelerated by GRAPPA) in volunteer studies. Moreover, higher temporal resolution improved the capture of flow features in IAS patients, highlighting the potential of CS-accelerated 4D flow MRI in neurovascular imaging (Liu et al., 2017). However, the sparse and random sampling of k-space in CS technology can lead to underestimation in flow and velocity data (Ma et al., 2019a). Therefore, the selection of AFs is crucial and requires achieving a delicate balance between clinical feasibility and maintaining image and data quality.

In the vascular area measurement, the overestimation of the area occurred in the high acceleration factor groups (CS ≥ 6) and the blood vessels with smaller diameter (SS). We speculated that the possible reason was the higher acceleration factor increased the noise of the image, and the negative impact of noise on the evaluation of smaller lumens was greater. The noise around the blood vessel was identified by the software as a false area.

No significant difference in the flow rate for all arteries and veins included in this study was observed among 4D flow MRI scans with different CS-AFs, compared to the reference scan. Gottwald et al. (2020) reported that stroke volumes and peak flow rate values from highly accelerated 4D flow MRI imaging (CS-AF = 20–30) were similar to those obtained with 2D flow MR imaging, providing context for our results. However, the mean velocity was significantly underestimated at the left ICA C2, right ICA C7, and SS by CS8 and CS10 groups, and at the right ICA C2 by the CS10 group compared to the SENSE4 group. Additionally, compared to the reference, peak velocity was significantly underestimated at the SS measured by the CS8 and CS10 groups. Furthermore, with the increase of AFs, the underestimation mentioned above became more pronounced. Similar underestimations of flow velocity have been described in previous studies using a CS-based acceleration approach (Harloff et al., 2019; Pathrose et al., 2020). Neuhaus et al. (2019) found no significant difference in flow parameters when the CS-AF was 6. However, as the AFs increased to 8 and 10, statistically significant underestimations were observed in major blood flow parameters such as net flow, peak flow, and peak velocity, similar to our findings. Building upon these observations, we further evaluated the impact of CS acceleration on WSS, including axial WSS, circumferential WSS, and 3D WSS, and found more severe underestimation compared to fundamental flow-derived parameters. To be specific, the following parameters were significantly underestimated compared to the reference: the average axial WSS at the right ICA C7 (measured by the CS8 group) and SS (measured by the CS8 and CS10 groups), the max axial WSS at the left TS (measured by the CS10 group) and SS (measured by the CS6-CS10 groups), and the 3D WSS at the left ICA C2 (measured by the CS6-10 groups), right ICA C2, C4, C7 (measured by the CS8 and CS10 groups), left TS, and SS (measured by the CS10 group). Similar to the underestimation of flow parameters, the higher the AFs, the greater the underestimation of WSS. Image-based WSS estimation is greatly affected by speed coding, speed resolution, and spatial resolution (Petersson et al., 2012). Peper et al. (2020) observed a slight decrease in mean WSS during peak systole for higher acceleration factors, with the decrease becoming more pronounced as AF increased. Pathrose et al. (2020) also noted the effect of CS on peak 3D WSS and speculated that the underestimation phenomenon of WSS is related to spatiotemporal undersampling. In our WSS analysis, we observed different performance between bilateral TSs at different AFs, with the hemodynamic parameters of the left TS appearing more susceptible to high acceleration. We speculate that in the case of the TSs, the right vessel was consistently dominant and easier to measure, while the left vessel was often smaller and exhibited flat, low flow rates—resulting in poor repeatability (Morgan et al., 2023). Larger lumen sizes typically lead to easier and more repeatable measurements for flow quantification, which is why the hemodynamic indexes of the SS are more likely to change under different AFs compared to other venous sinuses.

Using Bland–Altman plots, we observed good consistency and correlation between the two methods for conventional SENSE-accelerated and CS-accelerated 4D flow hemodynamic parameters. Whether it was flow derivative parameters such as flow rate and flow velocity or WSS, the hemodynamics measured by the CS4 group exhibited minimal bias and great limits of agreement compared to the reference in all blood vessels included in this study. With the increase of AFs, the consistency of the other groups decreased to varying degrees. The scan times for all 4D flow acquisitions in our study showed a wide range, attributed to changes in subjects’ heart rates over time and individual differences. For comparison and description purposes, we selected the scan time of 90 heart rates as an example to compare the differences in acquisition times between groups. The scan times for the SENSE4, CS4, CS6, CS8, and CS10 groups were 7 min and 24 s, 5 min and 47 s, 3 min and 52 s, 2 min and 54 s, and 2 min and 22 s, respectively. Compared to the SENSE4 group, the percentage reduction in scanning time of the CS-accelerated methods were 21.85, 47.75, 60.81, and 67.57% for CS4, CS6, CS8, and CS10, respectively. While high acceleration is advantageous for shortening the acquisition time, CS4 strikes a delicate balance between time-saving and high-quality hemodynamic measurements of both arteries and veins. Specifically, compared to the reference, there was no statistical difference in any hemodynamic parameters of any blood vessel with CS4. Therefore, CS4 could be a practical choice for routine clinical scanning.

Previous studies on the effect of spatial resolution of 4D blood flow MRI on aortic hemodynamic parameters have found that flow velocity and wall shear stress (WSS) are sensitive to changes in spatial resolution (Kweon et al., 2016; Montalba et al., 2018). El Sayed et al. (2023) found that when using 4D flow MRI to evaluate the hemodynamics of the ICA, compared to CFD, too low or too high isotropic spatial resolution will have a negative impact on velocity estimation. Low spatial resolution (0.5 mm isotropic) showed the presence of noise in the measurements, including velocity values outside the vessel lumen, while high spatial resolution (≥1.5 mm isotropic) blurred out the skewing of the velocity profile. Many studies in the literature have described an underestimation of WSS measurements acquired using 4D flow MRI (Potters W. et al., 2014; Potters W. V. et al., 2014; Cibis et al., 2016). The results show that the WSS values quantified by 4D flow MRI scan are underestimated by 12, 24, 45, and 73% compared to CFD at spatial resolutions of 0.74, 1.00, 1.50, and 2.00 mm^3^, respectively (El Sayed et al., 2023). Considering the balance between scanning time and image quality, we chose 1.6 mm isotropic for 4D flow MRI scanning, the reconstruction matrix size was varied to improve the spatial resolution (reconstruction voxel size = 0.8 mm isotropic), which basically meets the spatial resolution requirements of the above research. However, such voxel size seems to be still quite poor for the assessment of blood vessels with smaller lumens, such as the anterior cerebral artery and the posterior cerebral artery. In addition, we observed that high acceleration had a greater impact on smaller blood vessels in the process of image reconstruction, like the middle cerebral artery (MCA). When CS ≥ 6, most of the subjects had different degrees of truncation or deletion in the reconstructed MCA images. When calculating the 3D WSS, this effect became more obvious.

Current 4D flow MRI protocols are mainly based on measuring blood flow using a single predefined velocity encoding (VENC), as in our study, the 4D flow MRI scans were performed with two separate VENCs, one for arteries and the other for veins. However, this may lead to unexpectedly high velocity aliasing or high noise in slow flow rates and limit the ability of 4D flow MRI to fully capture the dynamic range of the velocity in the measured vessel (Schnell et al., 2016). The dual-VENC or multi-VENC methods proposed in recent years is an alternative method to solve this limitation, which uses a shared reference scan to obtain both low-VENC and high-VENC data. In this approach, high VENC data is used to correct the velocity aliasing in low VENC data to generate a single dual-venc dataset with a high velocity-to-noise ratio (Schnell et al., 2017). Mahinrad et al. (2022) showed excellent interobserver agreement for measurement of intracranial flow velocity using dual-VENC 4D flow MRI and demonstrated the feasibility of dual-VENC 4D flow MRI for quantitative assessment of intracranial hemodynamics and had moderate consistency with transcranial Doppler ultrasound. Compared to the high-VENC scan in arteries and the low-VENC scan in veins, the dual-VENC acquisition resulted in excellent correlation of net flow and peak velocity (Schnell et al., 2017). The current implementation requires 7 TRs for low VENC and high VENC data acquisition, which results in 75% longer scan time than a single VENC acquisition, but in applications where two 4D flow MRI scans at low-and high-venc are typically acquired, such as in the liver or brain, both venous and arterial blood flow are of interest, resulting in an overall scan time would actually be shorter (Schnell et al., 2017). Multi-VENC acquisition is optimized based on dual-VENC method, *in vivo* and vitro results indicate that triconditional triple-VENC algorithms proved superior to dual-VENC algorithms in correctly unwrapping aliased voxels, the quality of 4D flow MRI was further improved (Ma et al., 2019b).

As a prospective study, our research has several limitations. Firstly, it involves a small sample size, conducted at a single center, using a single MRI scanner, and employing a single post-processing software. Therefore, the applicability of our results needs verification in studies with larger sample sizes, across multiple centers, utilizing scanners from different manufacturers, and employing various post-processing software programs. Previous studies have reported differences in the repeatability and reproducibility of different 4D flow MRI post-processing software programs (Oechtering et al., 2023). And due to the inherent lack of spatial resolution of 4D flow MRI, our study only included arteries and venous sinuses with relatively large lumen for further analyses. Secondly, due to concerns about patient tolerance for long-term repeated scans, our study did not include patients. Future research should recruit individuals with relevant diseases to evaluate the applicability of CS-accelerated 4D flow MRI in pathological states. Thirdly, the four CS 4D flow scans were consistently acquired after the conventional SENSE-accelerated 4D flow acquisition. Changes in subjects’ heart rates and minor head movements due to intolerance to prolonged scanning may introduce bias to our results, which cannot be entirely avoided or controlled. In addition, we did not collect detailed heart rate data of the subjects during the examination, as well as some basic demographic data, such as blood pressure, body mass index (BMI). Fourthly, manual intervention is required by the operator for segmentation of blood vessels of interest, noise reduction, and placement of measurement planes due to the characteristics of post-processing software. Although we placed three measurement planes in each blood vessel to mitigate this issue to some extent, further efforts are needed to enhance the accuracy and reliability of measurement results. Finally, our study did not systematically analyze the effects of different temporal resolutions on CS 4D flow image quality or hemodynamics, despite evidence suggesting their impact on hemodynamic estimation, including WSS. Although the shortest repetition time allowed by the system with CS acceleration varies compared to traditional SENSE acceleration, we ensured that the methods we used were compared with the same temporal window to maintain consistency and minimize the potential impact of temporal resolution on our conclusions.

In conclusion, this study demonstrates that 4D flow MRI accelerated by CS4 of the cerebral artery and venous sinuses yielded data with excellent agreement compared to conventional SENSE-accelerated 4D flow MRI, with no statistical underestimation of hemodynamics observed in any vessel. Therefore, CS4 is recommended for non-invasive hemodynamics evaluation of both intracranial arteries and veins, serving as a high-throughput method that can replace parallel acquisition acceleration methods in routine clinical practice.

## Data availability statement

The raw data supporting the conclusions of this article will be made available by the authors, without undue reservation.

## Ethics statement

The studies involving humans were approved by the Ethics Committee of the First Affiliated Hospital of Dalian Medical University. The studies were conducted in accordance with the local legislation and institutional requirements. The participants provided their written informed consent to participate in this study.

## Author contributions

JC: Conceptualization, Data curation, Formal analysis, Investigation, Methodology, Project administration, Software, Visualization, Writing – original draft, Writing – review & editing. CY: Data curation, Formal analysis, Investigation, Methodology, Writing – review & editing. YZ: Data curation, Writing – review & editing. YQ: Data curation, Writing – review & editing. PC: Data curation, Writing – review & editing. JY: Data curation, Writing – review & editing. QS: Supervision, Writing – review & editing. YM: Resources, Supervision, Writing – review & editing.

## References

[ref1] Aoike T. Fujima N. Yoneyama M. Fujiwara T. Takamori S. Aoike S.. (2022). Development of three-dimensional MR neurography using an optimized combination of compressed sensing and parallel imaging. Magn. Reson. Imaging 87, 32–37. doi: 10.1016/j.mri.2021.12.002, PMID: 34968698

[ref2] Benjamin A. J. V. Bano W. Mair G. Thompson G. Casado A. Di Perri C.. (2020). Diagnostic quality assessment of IR-prepared 3D magnetic resonance neuroimaging accelerated using compressed sensing and k-space sampling order optimization. Magn. Reson. Imaging 74, 31–45. doi: 10.1016/j.mri.2020.08.025, PMID: 32890675

[ref3] Björnfot C. Garpebring A. Qvarlander S. Malm J. Eklund A. Wåhlin A. (2021). Assessing cerebral arterial pulse wave velocity using 4D flow MRI. J. Cereb. Blood Flow Metab. 41, 2769–2777. doi: 10.1177/0271678X211008744, PMID: 33853409 PMC8504412

[ref4] Boussel L. Rayz V. Martin A. Acevedo-Bolton G. Lawton M. T. Higashida R.. (2009). Phase-contrast magnetic resonance imaging measurements in intracranial aneurysms in vivo of flow patterns, velocity fields, and wall shear stress: comparison with computational fluid dynamics. Magn. Reson. Med. 61, 409–417. doi: 10.1002/mrm.21861, PMID: 19161132 PMC3055272

[ref5] Braig M. Menza M. Leupold J. LeVan P. Feng L. Ko C. W.. (2020). Analysis of accelerated 4D flow MRI in the murine aorta by radial acquisition and compressed sensing reconstruction. NMR Biomed. 33:e4394. doi: 10.1002/nbm.4394, PMID: 32815236

[ref6] Cibis M. Potters W. V. Gijsen F. J. Marquering H. van Ooij P. vanBavel E.. (2016). The effect of spatial and temporal resolution of cine phase contrast MRI on wall shear stress and oscillatory shear index assessment. PLoS One 11:e0163316. doi: 10.1371/journal.pone.0163316, PMID: 27669568 PMC5036833

[ref7] Demirkiran A. van Ooij P. Westenberg J. J. M. Hofman M. B. M. van Assen H. C. Schoonmade L. J.. (2022). Clinical intra-cardiac 4D flow CMR: acquisition, analysis, and clinical applications. Eur. Heart J. Cardiovasc. Imaging 23, 154–165. doi: 10.1093/ehjci/jeab112, PMID: 34143872 PMC8787996

[ref8] Ding H. Zhao P. Lv H. Li X. Qiu X. Zeng R.. (2021). Correlation between trans-stenotic blood flow velocity differences and the cerebral venous pressure gradient in transverse sinus stenosis: a prospective 4-dimensional flow magnetic resonance imaging study. Neurosurgery 89, 549–556. doi: 10.1093/neuros/nyab222, PMID: 34171923 PMC8440065

[ref9] El Sayed R. Sharifi A. Park C. C. Haussen D. C. Allen J. W. Oshinski J. N. (2023). Optimization of 4D flow MRI spatial and temporal resolution for examining complex hemodynamics in the carotid artery bifurcation. Cardiovasc. Eng. Technol. 14, 476–488. doi: 10.1007/s13239-023-00667-1, PMID: 37156900 PMC10524741

[ref10] Ferdian E. Marlevi D. Schollenberger J. Aristova M. Edelman E. R. Schnell S.. (2023). Cerebrovascular super-resolution 4D flow MRI-sequential combination of resolution enhancement by deep learning and physics-informed image processing to non-invasively quantify intracranial velocity, flow, and relative pressure. Med. Image Anal. 88:102831. doi: 10.1016/j.media.2023.102831, PMID: 37244143

[ref11] Garreau M. Puiseux T. Toupin S. Giese D. Mendez S. Nicoud F.. (2022). Accelerated sequences of 4D flow MRI using GRAPPA and compressed sensing: a comparison against conventional MRI and computational fluid dynamics. Magn. Reson. Med. 88, 2432–2446. doi: 10.1002/mrm.29404, PMID: 36005271 PMC9804839

[ref12] Gottwald L. M. Töger J. Markenroth Bloch K. Peper E. S. Coolen B. F. Strijkers G. J.. (2020). High spatiotemporal resolution 4D flow MRI of intracranial aneurysms at 7T in 10 minutes. Am. J. Neuroradiol. 41, 1201–1208. doi: 10.3174/ajnr.A6603, PMID: 32586964 PMC7357648

[ref13] Harloff A. Hagenlocher P. Lodemann T. Hennemuth A. Weiller C. Hennig J.. (2019). Retrograde aortic blood flow as a mechanism of stroke: MR evaluation of the prevalence in a population-based study. Eur. Radiol. 29, 5172–5179. doi: 10.1007/s00330-019-06104-z 30877458

[ref14] Jaeger E. Sonnabend K. Schaarschmidt F. Maintz D. Weiss K. Bunck A. C. (2020). Compressed-sensing accelerated 4D flow MRI of cerebrospinal fluid dynamics. Fluids Barriers CNS 17:43. doi: 10.1186/s12987-020-00206-3, PMID: 32677977 PMC7364783

[ref15] Kazemi A. Padgett D. A. Callahan S. Stoddard M. Amini A. A. (2022). Relative pressure estimation from 4D flow MRI using generalized Bernoulli equation in a phantom model of arterial stenosis. Magn. Reson. Mater. Phys. 35, 733–748. doi: 10.1007/s10334-022-01001-x 35175449

[ref16] Kilinc O. Chu S. Baraboo J. Weiss E. K. Engel J. Maroun A.. (2022). Hemodynamic evaluation of type B aortic dissection using compressed sensing accelerated 4D flow MRI. J. Magn. Reson. Imaging 57, 1752–1763. doi: 10.1002/jmri.28432, PMID: 36148924 PMC10033465

[ref17] Kim D. Heo Y. J. Jeong H. W. Baek J. W. Shin G. W. Jin S-C.. (2021). Compressed sensing time-of-flight magnetic resonance angiography with high spatial resolution for evaluating intracranial aneurysms: comparison with digital subtraction angiography. Neuroradiol. J. 34, 213–221. doi: 10.1177/1971400920988099, PMID: 33455533 PMC8165900

[ref18] Ko S. Yang B. Cho J. H. Lee J. Song S. (2019). Novel and facile criterion to assess the accuracy of WSS estimation by 4D flow MRI. Med. Image Anal. 53:95. doi: 10.1016/j.media.2019.01.009, PMID: 30743192

[ref19] Kweon J. Yang D. H. Kim G. B. Kim N. Paek M. Y. Stalder A. F.. (2016). Four-dimensional flow MRI for evaluation of post-stenotic turbulent flow in a phantom: comparison with flowmeter and computational fluid dynamics. Eur. Radiol. 26, 3588–3597. doi: 10.1007/s00330-015-4181-6, PMID: 26747263

[ref20] Li Y. Amili O. Moen S. van de Moortele P. F. Grande A. Jagadeesan B.. (2022). Flow residence time in intracranial aneurysms evaluated by in vitro 4D flow MRI. J. Biomech. 141:111211. doi: 10.1016/j.jbiomech.2022.111211, PMID: 35780698

[ref21] Li X. Qiu X. Ding H. Lv H. Zhao P. Yang Z.. (2021). Effects of different morphologic abnormalities on hemodynamics in patients with venous pulsatile tinnitus: a four-dimensional flow magnetic resonance imaging study. J. Magn. Reson. Imaging 53, 1744–1751. doi: 10.1002/jmri.27503, PMID: 33491233 PMC8248416

[ref22] Liu J. Koskas L. Faraji F. Kao E. Wang Y. Haraldsson H.. (2017). Highly accelerated intracranial 4D flow MRI: evaluation of healthy volunteers and patients with intracranial aneurysms. Magn. Reson. Mater. Phys. 31, 295–307. doi: 10.1007/s10334-017-0646-8, PMID: 28785850 PMC5803461

[ref23] Ma L. E. Markl M. Chow K. Huh H. Forman C. Vali A.. (2019a). Aortic 4D flow MRI in 2 minutes using compressed sensing, respiratory controlled adaptive k-space reordering, and inline reconstruction. Magn. Reson. Med. 81, 3675–3690. doi: 10.1002/mrm.27684, PMID: 30803006 PMC6535305

[ref24] Ma L. E. Markl M. Chow K. Vali A. Wu C. Schnell S. (2019b). Efficient triple-VENC phase-contrast MRI for improved velocity dynamic range. Magn. Reson. Med. 83, 505–520. doi: 10.1002/mrm.27943, PMID: 31423646 PMC7051107

[ref25] Mahinrad S. Tan C. O. Ma Y. Aristova M. Milstead A. L. Lloyd-Jones D.. (2022). Intracranial blood flow quantification by accelerated dual-venc 4D flow MRI: comparison with transcranial Doppler ultrasound. J. Magn. Reson. Imaging 56, 1256–1264. doi: 10.1002/jmri.28115, PMID: 35146822 PMC9363520

[ref26] Marlevi D. Schollenberger J. Aristova M. Ferdian E. Ma Y. Young A. A.. (2021a). Noninvasive quantification of cerebrovascular pressure changes using 4D flow MRI. Magn. Reson. Med. 86, 3096–3110. doi: 10.1002/mrm.28928, PMID: 34431550 PMC11421438

[ref27] Marlevi D. Sotelo J. A. Grogan-Kaylor R. Ahmed Y. Uribe S. Patel H. J.. (2021b). False lumen pressure estimation in type B aortic dissection using 4D flow cardiovascular magnetic resonance: comparisons with aortic growth. J. Cardiovasc. Magn. Reson. 23:51. doi: 10.1186/s12968-021-00741-4, PMID: 33980249 PMC8117268

[ref28] Meister R. L. Groth M. Zhang S. Buhk J. H. Herrmann J. (2023). Evaluation of artifact appearance and burden in pediatric brain tumor MR imaging with compressed sensing in comparison to conventional parallel imaging acceleration. J. Clin. Med. 12:5732. doi: 10.3390/jcm12175732, PMID: 37685799 PMC10489124

[ref29] Miller K. B. Howery A. J. Rivera-Rivera L. A. Johnson S. C. Rowley H. A. Wieben O.. (2019). Age-related reductions in cerebrovascular reactivity using 4D flow MRI. Front. Aging Neurosci. 11:281. doi: 10.3389/fnagi.2019.00281, PMID: 31680935 PMC6811507

[ref30] Moersdorf R. Treutlein M. Kroeger J. R. Ruijsink B. Wong J. Maintz D.. (2019). Precision, reproducibility and applicability of an undersampled multi-venc 4D flow MRI sequence for the assessment of cardiac hemodynamics. Magn. Reson. Imaging 61:73. doi: 10.1016/j.mri.2019.05.015, PMID: 31100318

[ref31] Montalba C. Urbina J. Sotelo J. Andia M. E. Tejos C. Irarrazaval P.. (2018). Variability of 4D flow parameters when subjected to changes in MRI acquisition parameters using a realistic thoracic aortic phantom. Magn. Reson. Med. 79, 1882–1892. doi: 10.1002/mrm.26834, PMID: 28714282

[ref32] Morgan A. G. Thrippleton M. J. Stringer M. Jin N. Wardlaw J. M. Marshall I. (2023). Repeatability and comparison of 2D and 4D flow MRI measurement of intracranial blood flow and pulsatility in healthy individuals and patients with cerebral small vessel disease. Front. Psychol. 14:1125038. doi: 10.3389/fpsyg.2023.1125038, PMID: 37325748 PMC10262051

[ref33] Neuhaus E. Weiss K. Bastkowski R. Koopmann J. Maintz D. Giese D. (2019). Accelerated aortic 4D flow cardiovascular magnetic resonance using compressed sensing: applicability, validation and clinical integration. J. Cardiovasc. Magn. Reson. 21:65. doi: 10.1186/s12968-019-0573-0, PMID: 31638997 PMC6802342

[ref34] Oechtering T. H. Nowak A. Sieren M. M. Stroth A. M. Kirschke N. Wegner F.. (2023). Repeatability and reproducibility of various 4D flow MRI postprocessing software programs in a multi-software and multi-vendor cross-over comparison study. J. Cardiovasc. Magn. Reson. 25:22. doi: 10.1186/s12968-023-00921-4, PMID: 36978131 PMC10052852

[ref35] Panayiotou H. R. Mills L. K. Broadbent D. A. Shelley D. Scheffczik J. Olaru A. M.. (2022). Comprehensive neonatal cardiac, feed and wrap, non-contrast, non-sedated, free-breathing compressed sensing 4D flow MRI assessment. J. Magn. Reson. Imaging 57, 789–799. doi: 10.1002/jmri.28325, PMID: 35792484

[ref36] Park S. Kwon M. Nam H. Huh H. (2023). Interpolation time-optimized aortic pulse wave velocity estimation by 4D flow MRI. Sci. Rep. 13:16484. doi: 10.1038/s41598-023-43799-z, PMID: 37777620 PMC10542805

[ref37] Pathrose A. Ma L. Berhane H. Scott M. B. Chow K. Forman C.. (2020). Highly accelerated aortic 4D flow MRI using compressed sensing: performance at different acceleration factors in patients with aortic disease. Magn. Reson. Med. 85, 2174–2187. doi: 10.1002/mrm.28561, PMID: 33107141 PMC7846046

[ref38] Peper E. S. Gottwald L. M. Zhang Q. Coolen B. F. van Ooij P. Nederveen A. J.. (2020). Highly accelerated 4D flow cardiovascular magnetic resonance using a pseudo-spiral Cartesian acquisition and compressed sensing reconstruction for carotid flow and wall shear stress. J. Cardiovasc. Magn. Reson. 22:7. doi: 10.1186/s12968-019-0582-z, PMID: 31959203 PMC6971939

[ref39] Perez-Raya I. Fathi M. F. Baghaie A. Sacho R. D’Souza R. M. (2021). Modeling and reducing the effect of geometric uncertainties in intracranial aneurysms with polynomial Chaos expansion, data decomposition, and 4D-flow MRI. Cardiovasc. Eng. Technol. 12, 127–143. doi: 10.1007/s13239-020-00511-w, PMID: 33415699

[ref40] Petersson S. Dyverfeldt P. Ebbers T. (2012). Assessment of the accuracy of MRI wall shear stress estimation using numerical simulations. J. Magn. Reson. Imaging 36, 128–138. doi: 10.1002/jmri.23610, PMID: 22336966

[ref41] Potters W. Marquering H. Van Bavel E. Nederveen A. J. (2014). Measuring wall shear stress using velocity-encoded MRI. Curr. Cardiovasc. Imaging 7, 1–12. doi: 10.1007/s12410-014-9257-1

[ref42] Potters W. V. van Ooij P. Marquering H. vanBavel E. Nederveen A. J. (2014). Volumetric arterial wall shear stress calculation based on cine phase contrast MRI. J. Magn. Reson. Imaging 41, 505–516. doi: 10.1002/jmri.24560, PMID: 24436246

[ref43] Schnell S. Ansari S. A. Wu C. Garcia J. Murphy I. G. Rahman O. A.. (2017). Accelerated dual-venc 4D flow MRI for neurovascular applications. J. Magn. Reson. Imaging 46, 102–114. doi: 10.1002/jmri.25595, PMID: 28152256 PMC5464980

[ref44] Schnell S. Wu C. Ansari S. A. (2016). Four-dimensional MRI flow examinations in cerebral and extracerebral vessels – ready for clinical routine? Curr. Opin. Neurol. 29, 419–428. doi: 10.1097/WCO.0000000000000341, PMID: 27262148 PMC4939804

[ref45] Schuchardt F. F. Kaller C. P. Strecker C. Lambeck J. Wehrum T. Hennemuth A.. (2019). Hemodynamics of cerebral veins analyzed by 2D and 4D flow MRI and ultrasound in healthy volunteers and patients with multiple sclerosis. J. Magn. Reson. Imaging 51, 205–217. doi: 10.1002/jmri.26782, PMID: 31102341

[ref46] Sodhi A. Markl M. Popescu A. R. Griffin L. M. Robinson J. D. Rigsby C. K. (2023). Highly accelerated compressed sensing 4D flow MRI in congenital and acquired heart disease: comparison of aorta and main pulmonary artery flow parameters with conventional 4D flow MRI in children and young adults. Pediatr. Radiol. 53:2597, –2607. doi: 10.1007/s00247-023-05788-2 37882844

[ref47] Sui H. Gong Y. Liu L. Lv Z. Zhang Y. Dai Y.. (2023). Comparison of artificial intelligence-assisted compressed sensing (ACS) and routine two-dimensional sequences on lumbar spine imaging. J. Pain Res. 16, 257–267. doi: 10.2147/JPR.S388219, PMID: 36744117 PMC9891076

[ref48] Vollherbst D. F. Bendszus M. Möhlenbruch M. A. (2018). Intracranial vascular malformations. Nervenarzt 89, 1179–1194. doi: 10.1007/s00115-018-0606-1 30215133

[ref49] Xie L. Zhang Y. Hong H. Xu S. Cui L. Wang S.. (2024). Higher intracranial arterial pulsatility is associated with presumed imaging markers of the glymphatic system: an explorative study. NeuroImage 288:120524. doi: 10.1016/j.neuroimage.2024.120524, PMID: 38278428

[ref50] Zhang Y. Cao J. Qiao C. Gao B. Du W. Lin L.. (2024). Fast imaging of lenticulostriate arteries by high-resolution black-blood T1-weighted imaging with variable flip angles and acceleration by compressed sensitivity encoding. Magn. Reson. Imaging 110, 51–56. doi: 10.1016/j.mri.2024.03.004, PMID: 38458551

[ref51] Zhang G. Wang Z. Zhang S. Qin Y. Yao Y. Tang X.. (2020). Age and anatomical location related hemodynamic changes assessed by 4D flow MRI in the carotid arteries of healthy adults. Eur. J. Radiol. 128:109035. doi: 10.1016/j.ejrad.2020.109035, PMID: 32413676

[ref52] Zhang G. Zhang S. Qin Y. Fang J. Tang X. Li L.. (2021). Differences in wall shear stress between high-risk and low-risk plaques in patients with moderate carotid artery stenosis: a 4D flow MRI study. Front. Neurosci. 15:678358. doi: 10.3389/fnins.2021.678358, PMID: 34456667 PMC8385133

